# In vitro assessment of 17 antimicrobial agents against clinical *Mycobacterium avium* complex isolates

**DOI:** 10.1186/s12866-022-02582-2

**Published:** 2022-07-08

**Authors:** Siran Lin, Wenya Hua, Shiyong Wang, Yu Zhang, Xinchang Chen, Hong Liu, Lingyun Shao, Jiazhen Chen, Wenhong Zhang

**Affiliations:** 1 Department of Infectious Diseases, Shanghai Key Laboratory of Infectious Diseases and Biosafety Emergency Response, National Medical Center for Infectious Diseases, Huashan Hospital, Fudan University, Shanghai, China; 2 Department of Laboratory Medicine, Huashan Hospital, Fudan University, Shanghai, China; 3 National Clinical Research Center for Aging and Medicine, Huashan Hospital, Fudan University, Shanghai, 200040 China; 4 State Key Laboratory of Genetic Engineering, School of Life Science, Fudan University, Shanghai, 200438 China; 5 Key Laboratory of Medical Molecular Virology (MOE/MOH) and Institutes of Biomedical Sciences, Shanghai Medical College, Fudan University, Shanghai, 200032 China

**Keywords:** *Mycobacterium avium* complex (MAC), Drug susceptibility test, Minimum inhibitory concentration (MIC), *Mycobacterium intracellulare*, *Mycobacterium avium*

## Abstract

**Background:**

Recently, *Mycobacterium avium* complex (MAC) infections have been increasing, especially in immunocompromised and older adults. The rapid increase has triggered a global health concern due to limited therapeutic strategies and adverse effects caused by long-term medication. To provide more evidence for the treatment of MAC, we studied the in vitro inhibitory activities of 17 antimicrobial agents against clinical MAC isolates.

**Results:**

A total of 111 clinical MAC isolates were enrolled in the study and they were identified as *M. intracellulare*, *M. avium*, *M. marseillense*, *M. colombiense*, *M. yongonense*, and two isolates could not be identified at the species level. MAC strains had relatively low (0–21.6%) resistance to clarithromycin, amikacin, bedaquiline, rifabutin, streptomycin, and clofazimine, and the resistant rates to isoniazid, rifampin, linezolid, doxycycline, and ethionamide were very high (72.1–100%). In addition, *M. avium* had a significantly higher resistance rate than that of *M. intracellulare* for ethambutol (92.3% vs 40.7%, *P* < 0.001), amikacin (15.4% vs 1.2%, *P* = 0.049), and cycloserine (69.2% vs 25.9%, *P* = 0.004).

**Conclusions:**

Our results supported the current usage of macrolides, rifabutin, and aminoglycosides in the regimens for MAC infection, and also demonstrated the low resistance rate against new drugs, such as clofazimine, tedizolid, and bedaquiline, suggesting the possible implementation of these drugs in MAC treatment.

**Supplementary Information:**

The online version contains supplementary material available at 10.1186/s12866-022-02582-2.

## Background

Members of the *Mycobacterium avium* complex (MAC) are the most common nontuberculous mycobacteria (NTM) species that cause pulmonary, soft tissue, and systemic diseases. MAC tends to cause infection in people with immunodeficiencies or underlying lung diseases. Host factors associated with MAC infection include acquired immunodeficiency syndrome, gene mutations in the interferon gamma (IFN-γ)-interleukin 12 axis, positive anti-IFN-γ autoantibodies, cystic fibrosis, and bronchiectasis [1–3]. Over the last decade, the incidence of MAC infections has increased, along with the emergence of several novel species. After 2015, *Mycobacterium intracellulare* has become the most prevalent NTM species in China instead of *Mycobacterium abscessus*, according to a meta-analysis in 2020 [4]. *M. intracellulare* and *Mycobacterium avium* remain the most important and prevalent pathogens in the MAC [5], while other species, including *Mycobacterium chimaera *[6], *Mycobacterium colombiense *[7], and *Mycobacterium marseillense *[8] in the MAC have been increasingly reported recently.

*M. chimaera*, one of the species of *M. intracellulare*, is transmitted through contaminated catheters and often causes disseminated and life-threatening infections in people who have undergone open-heart surgery [9, 10]. As for *M. colombiense* and *M. marseillense*, they are genetically different from *M. avium* and *M. intracellulare *[11, 12]*. M. colombiense* was first reported in Columbian patients with human immunodeficiency virus [13], and has since been isolated from both immunocompromised and immunocompetent patients with cutaneous, lymph node, and pulmonary infections [14–16]. *M. marseillense*, which was identified later in 2009, has similar pathogenicity to *M. colombiense*. For Other species, like *Mycobacterium vulneris*, *Mycobacterium timonense*, *Mycobacterium arosiense*, *Mycobacterium yongonense*, and *Mycobacterium bouchedurhonense*, few cases were reported.

MAC infections can be difficult to treat due to multiple factors, including environmental and genetic risk factors and frequent drug-related side effects. A culture conversion rate of 50%-80%, a recurrence rate of 25%-48%, and a reinfection rate of 46%-75% have been observed in patients with MAC lung diseases (MAC-LD) [17–19]. Treatment guidelines for MAC-LD by the American Thoracic Society and the British Thoracic Society recommended a three-drug therapeutic approach that includes macrolides, rifampin, and ethambutol [20]. Additionally, for patients with refractory, severe or macrolide-resistant MAC-LD, parenteral amikacin or streptomycin are recommended treatments. In the MAC treatment regimen, only macrolides and amikacin undergo drug susceptibility testing [21–23], as the other agents lack correlations between in vitro testing and in vivo clinical response. Recently, a limited number of new antibiotics, including anti-tuberculous agents, such as clofazimine [24], has been introduced to treat MAC.

Although in vitro drug susceptibility testing of MAC is routine, novel drugs are rarely tested. In addition, the prevalent MAC species differ by regions, which could cause different resistance profiles of MAC from different regions. Therefore, we conducted species identification and drug susceptibility testing on the MAC strains collected from patients admitted to our hospital in Shanghai, China. In addition to the frequently used drugs, we also tested clofazimine, bedaquiline, tedizolid, and cycloserine, with the aim of exploring the effectiveness of antimicrobials against MAC, Because they are new accessible drugs and they are recommended for treating tuberculosis by WHO, except for tedizolid. It suggests that they have the potential to be developed as anti-NTM drugs, with certain safety and tolerance. In addition, clinical trials and vitro experiments have been conducted to study the therapeutic efficacy of these drugs on NTM diseases [25–29].

## Results

**Fig. 1:**
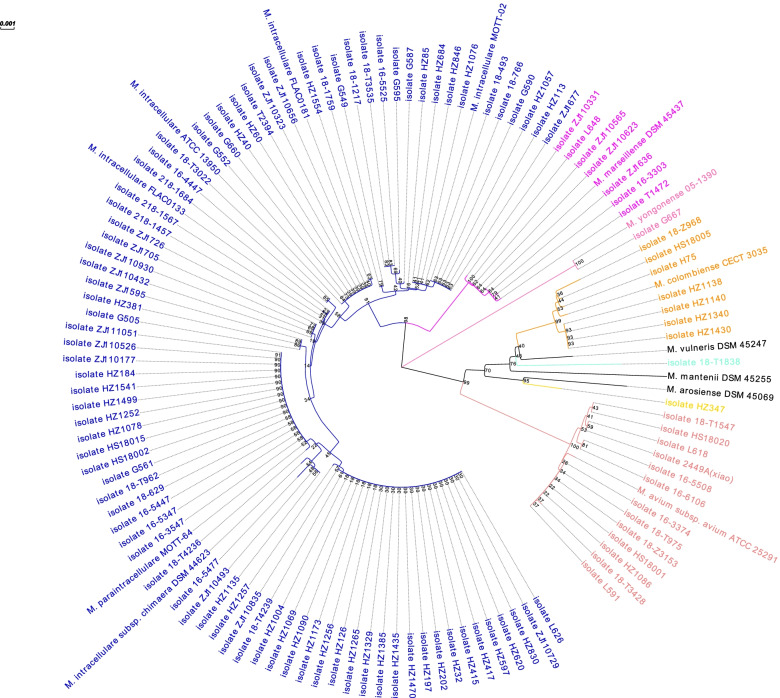
Phylogenetic analyses of concatenated *hsp65* and *rpoB* gene sequences of clinical MAC isolates, using the neighbor-joining method by MEGA10 software. The bootstrap value marked on the node is used to evaluate the reliability of the branch. The evolutionary branch length value on the branch indicates the genetic variability of the evolutionary branch. Each species is marked with the same color: *M. intracellulare* (medium blue), *M. avium* (light coral), *M. marseillense* (fuchsia), *M. colombiense* (dark orange), *M. yongonense* (hot pink), isolate HZ347 (gold), and isolate 18-T1838 (aquamarine)

A total of 111 MAC isolates were collected and were identified as *M. intracellulare* (*n* = 81), *M. avium* (*n* = 13), *M. marseillense* (*n* = 7), *M. colombiense* (*n* = 7), *M. yongonense* (*n* = 1) by the criteria that the similarity on concatenated *hsp65* and *rpoB* gene sequences was greater than 99.3% between type strains and the clinical isolates [30] (Fig. 1). The similarity between HZ347 and the *M. arosiense* type strain was 98.94%. Similarly, the isolate 18-T1838 and the *M. vulneris* type strain was the most closely related and they shared 98.8% coincidence in concatenated *hsp65* and *rpoB* gene. Therefore, the species of isolate HZ347 and 18-T1838 cannot be confirmed.

We tested the antimicrobial activities of 17 antimicrobial agents against 111 MAC isolates. The results were showed in Table 1 and Table 2. The detailed MIC values of different species were listed in Supplementary Table 1. The MAC isolates showed a low resistance rate to commonly used drugs, such as clarithromycin (4.5%, 5/111), amikacin (2.7%, 3/111), rifabutin (21.6%, 24/111), and streptomycin (17.1%, 19/111). However, they were highly resistant to most anti-tuberculosis drugs, such as isoniazid (100%, 111/111), rifampin (82.9%, 92/111), linezolid (72.1%, 80/111), doxycycline (98.2%, 109/111), and ethionamide (91.9%, 102/111). Specifically, all the MAC isolates were resistant to isoniazid. Besides, ciprofloxacin also showed a poor inhibitory effect on MAC isolates which had a resistance rate of 87.4% (97/111). Furthermore, the MAC isolates showed an intermediate resistance rate for ethambutol (54.1%, 60/111), trimethoprim/sulfamethoxazole (62.2%, 69/111), and moxifloxacin (60.4%, 67/111). Interestingly, the MAC isolates showed a low resistance rate for all four newly used drugs: bedaquiline (0%, 0/111), clofazimine (19.8%, 22/111), tedizolid (26.1%, 29/111), and cycloserine (30.6%, 34/111).

**Table 1:** Drug resistant rates of different MAC species

Antimicrobial agent	No. of resistant isolates (%)								*P* value
	**All isolates** ***n* = 111**	***M. intracellulare*** ***n* = 81**	***M. avium*** ***n* = 13**	***M. marseillense*** ***n* = 7**	***M. colombiense*** ***n* = 7**	***M. yongonense*** ***n* = 1**	**HZ347** ***n* = 1**	**18-T1838** ***n* = 1**	
CLA	5(4.5%)	3(3.7%)	2(15.4%)	0(0%)	0(0%)	0	0	0	0.139
RFB	24(21.6%)	17(21.0%)	2(15.4%)	1(14.3%)	3(42.9%)	0	1	0	> 0.999
EMB	60(54.1%)	33(40.7%)	12(92.3%)	6(85.7%)	7(100%)	1	1	0	< 0.001
INH	111(100%)	81(100%)	13(100%)	7(100%)	7(100%)	1	1	1	> 0.999
MXF	67(60.4%)	43(53.1%)	8(61.5%)	6(85.7%)	7(100%)	1	1	1	0.570
RIF	92(82.9%)	64(79.0%)	11(84.6%)	7(100%)	7(100%)	1	1	1	> 0.999
SXT	69(62.2%)	45(55.6%)	10(76.9%)	6(85.7%)	5(71.4%)	1	1	1	0.226
AMI	3(2.7%)	1(1.2%)	2(15.4%)	0(0%)	0(0%)	0	0	0	0.049
LZD	80(72.1%)	53(65.4%)	11(84.6%)	7(100%)	6(85.7%)	1	1	1	0.213
CIP	97(87.4%)	70(86.4%)	10(76.9%)	7(100%)	7(100%)	1	1	1	0.404
STR	19(17.1%)	17(21.0%)	2(15.4%)	0(0%)	0(0%)	0	0	0	> 0.999
DOX	109(98.2%)	79(97.5%)	13(100%)	7(100%)	7(100%)	1	1	1	> 0.999
ETH	102(91.9%)	75(92.6%)	10(76.9%)	7(100%)	7(100%)	1	1	1	0.107
TZD	29(26.1%)	24(29.6%)	4(30.8%)	0(0%)	1(14.3%)	0	0	0	> 0.999
CFZ	22(19.8%)	14(17.3%)	4(30.8%)	2(28.6%)	2(28.6%)	0	0	0	0.265
BDQ	0(0%)	0(0%)	0(0%)	0(0%)	0(0%)	0	0	0	> 0.999
CS	34(30.6%)	21(25.9%)	9(69.2%)	1(14.3%)	2(28.6%)	1	0	0	0.004

Notes: The *P* value represent comparisons between *M. intracellulare* and *M. avium*

**Table 2:** MIC_50_ and MIC_90_ values of *M. intracellulare* and *M. avium*

Antimicrobial agent	*M. intracellulare* (*n* = 81) MIC (μg/mL)			*M. avium* (*n* = 13) MIC (μg/mL)		
	**Range**	**50%**	**90%**	**Range**	**50%**	**90%**
CLA	≤ 0.06 to > 64	4	8	1–16	4	16
RFB	≤ 0.25 to > 8	2	4	≤ 0.25–4	1	4
EMB	1 to > 16	4	> 16	4 to > 16	16	> 16
INH	2 to > 8	> 8	> 8	2 to > 8	> 8	> 8
MXF	≤ 0.12 to > 8	4	8	0.5 to > 8	> 8	> 8
RIF	≤ 0.12 to > 8	8	> 8	4 to > 8	8	> 8
SXT	≤ 0.12/2.38 to > 8/152	4/76	> 8/152	2/38 to > 8/152	> 8/152	> 8/152
AMI	≤ 1 to > 64	8	16	2–32	4	16
LZD	≤ 1 to > 64	32	64	4–64	32	64
CIP	≤ 0.12 to > 16	16	> 16	1 to > 16	> 16	> 16
STR	≤ 0.5 to > 64	16	64	2–32	16	32
DOX	2 to > 16	> 16	> 16	> 16	> 16	> 16
ETH	2.5 to > 20	> 20	> 20	5 to > 20	> 20	> 20
TZD	≤ 0.5 to > 32	8	16	1 to > 32	8	32
CFZ	≤ 0.25 to > 8	2	8	1–8	2	4
BDQ	0.015–0.12	0.06	0.12	0.03–0.12	0.06	0.12
CS	8 to > 64	32	64	16–64	32	64

Most agents showed similar antimicrobial activities against the two main MAC species, *M. intracellulare* and *M. avium*. However, *M. avium* had a higher resistance rate than that of *M. intracellulare* for clarithromycin (15.4%, 2/13 vs 3.7%, 3/81), ethambutol (92.3%, 12/13 vs 40.7%, 33/81), trimethoprim/sulfamethoxazole (76.9%, 10/13 vs 55.6%, 45/81), amikacin (15.4%, 2/13 vs 1.2%, 1/81), linezolid (84.6%,11/13 vs 65.4%,53/81), clofazimine (30.8%, 4/13 vs 17.3%,14/81), and cycloserine (69.2%, 9/13 vs 25.9%, 21/81)*. M. intracellulare* had a higher resistance to ethionamide than *M. avium*. The differences in the resistance rates of amikacin, ethambutol, and cycloserine were statistically significant (*P* = 0.049, *P* < 0.001, and *P* = 0.004, respectively). All or almost all the *M. marseillense* and *M. colombiense* isolates were resistant to ethambutol, isoniazid, moxifloxacin, rifampin, trimethoprim/sulfamethoxazole, linezolid, ciprofloxacin, doxycycline, and ethionamide, while none of them were resistant to clarithromycin, amikacin, streptomycin, or bedaquiline (Table 1). The other agents showed good inhibitory activities against the two species which had a resistance rate ranging from 0% to 42.9% (3/7). *M. yongonense*, *M. arosience*, isolate HZ347, and isolate 18-T1838 had similar resistance profiles against the 17 antimicrobial agents, except for that *M. yongonense* was resistant to cycloserine (MIC > 64 μg/mL) and isolate HZ347 was resistant to rifabutin.

## Discussion

Our antibiotic susceptibility testing results supported the current recommendation of using macrolides, rifamycins, and aminoglycosides to treat MAC infections. The medium for MIC measurement was changed to 7H9 with 10% OADC due to poor growth in cation-adjusted Muller Hinton Broth (CAMHB). According to a study in 2020, the drug susceptibility testing for MAC in 7H9 is found easier for measurement and has greater reproducibility compared with CAMHB [31]. The breakpoints of rifabutin, rifampin, trimethoprim/sulfamethoxazole, ciprofloxacin, and doxycycline for *M.kansasii*, and the breakpoints of ethambutol, isoniazid, and ethionamide for *M.tuberculosis* were used on the MAC isolates. They have similar cellular structure and share similar niches in the mononuclear phagocyte system in vivo. Therefore, we thought it is acceptable to use breakpoints for *M.kansasii* and *M.tuberculosis* in MAC isolates. And it is common to use the same breakpoints for different NTM species in previous studies due to insufficient information about drug breakpoints for each NTM species [29, 32].

In our study, clarithromycin showed good inhibitory activity against all MAC isolates, consistent with previous studies [33, 34]. We performed the 23S rRNA gene sequencing on the clarithromycin-resistant isolates, and found that two strains with MIC greater than 64 μg/ml had known mutation in the 23S rRNA (data not shown). The resistance rate of MAC isolates against rifampin was 82.9% (92/111), which was in agreement (78.9%; 216/274) with a previous study [35]. Unlike rifampin, rifabutin showed a better antimicrobial activity and was recommended as an alternative to rifampicin, especially for disseminated MAC infections, for patients infected with MAC [36]. However, in a recent study, neither rifampin or rifabutin inhibited MAC growth in vitro [37]. Therefore, further clinical trials are still needed to determine the best choice among different rifamycins for treating MAC diseases. The intermediate resistance against ethambutol was comparable with that of a previous study (58.1%;159/274) [35]. These results do not support the usage of ethambutol for MAC. Among the aminoglycosides, amikacin may be better for treating MAC infections than streptomycin, with an overall low resistant rate of 2.7% (24/111), which is as low as shown in previous studies [38, 39]. No common mutations were found in the *rrs* gene of the four amikacin-resistant isolates (data not shown). Streptomycin is a potentially good choice for treatment of MAC isolates. In a study in a Taiwanese district, the resistance rate of MAC isolates against streptomycin was even lower (4.8%; 4/83) [29]. This difference may be regional (different geographies) or may be due to the inconsistent proportions of MAC species collected in the studies.

As second-line drugs for MAC disease, the clinical efficacy of moxifloxacin and linezolid remains uncertain [40]. In our study, both had limited activity against MAC isolates, which is comparable with previous studies in Korea [41], Sweden [39], and China [42]. However, unlike the poor activity in vitro, a recent study has shown that fluoroquinolone-containing regimens could achieve similar clinical improvement with the standard regimen and could be an alternative for patients who cannot tolerate the standard regimen [43]. As for the other tested anti-tuberculosis drugs, such as isoniazid, ciprofloxacin, doxycycline, and ethionamide, the MAC isolates showed high resistance, which supported the consensus that these drugs should not be used in the treatment of MAC diseases as shown in a previous study [34]. The comparison of drug resistance rate of recommended agents for MAC isolates from different studies were shown in Table 3.

**Table 3:** The comparison of drug resistance rate of recommended agents for MAC isolates from different studies

NATION/DISTRICT	YEAR	ISOLATE	CLA	RIF	EMB	MXF	RFB	AMI	LZD	STR	SOURCE
THIS STUDY	2021	111	5(4.5%)	92(82.9%)	60(54.1%)	67(60.4%)	24(21.6%)	3(2.7%)	80(72.1%)	19(17.1%)	
GERMANY	2020	98	1(1.2%)	-	-	38(44.7%)	-	0(0%)	57(67.1%)	-	[33]
GERMANY	2019	683	17(2.5%)	-	-	430(63.1%)	-	-	511(75.0%)	-	[34]
KOREA	2018	1883	95 (5.0%)	1080 (57.4%)	1691 (89.8%)	1054 (56.0%)	-	166 (8.8%)	805 (42.8%)	-	[41]
SWEDEN	2017	229	6 (2.6%)	210 (91.7%)	-	112 (48.9%)	-	11 (4.8%)	118 (51.5%)	-	[39]
TAIWAN	2018	83	0(0%)	-	-	72(86.7%)	-	2(2.4%)	61(73.5%)	4(4.8%)	[29]
UK	2016	-	248(19.9%)	686(55.7%)	391(31.9%)	-	58(5.9%)	100(8.2%)	-	498(53.0%)	[55]

In our study, the new oxazolidinone, tedizolid, had a significantly lower resistance rate than linezolid, supporting the previous results which indicated that tedizolid has enhanced in vitro activities against several NTM species [44]. In addition, it has less side effects in long-term therapy, compared with linezolid and has a concentration-dependent activity against *M. avium*. Its efficacy can be enhanced by ethambutol, which suggests its potential role in the treatment of MAC diseases [45].

Clofazimine, which also had a low resistance rate in our study, has been recently proven to be an effective agent for the treatment of MAC both in patients and mouse models [46, 47]. A recent study conducted in Korea found that a lower MIC value of clofazimine (≤ 0.25 mg/L) was associated with negative conversion of sputum culture in patients with NTM lung diseases [26]. Another study in Korea demonstrated that clofazimine, together with inhaled amikacin, could provide favorable outcomes in patients with refractory MAC-LD [25]. Nevertheless, the adverse effects of clofazimine are a major concern that affects its application in patients.

Bedaquiline is a diarylquinoline antibiotic, acting through an antimicrobial mechanism by inhibiting F1Fo-ATP synthase, an enzyme that is essential in *Mycobacterium tuberculosis *[48]*.* Although several clinical studies have found increased sputum conversion rates with bedaquiline in patients with multidrug-resistant tuberculosis, its efficacy in the treatment of MAC-LD is currently controversial. In some studies, bedaquiline is considered to be a good candidate for refractory or relapsing diseases caused by MAC [27, 49], while in other studies, bedaquiline treatment in patients with MAC-LD were not favorable due to the emergence of resistance and the decreased systemic exposure caused by rifamycin through the induction of cytochrome P450 [50, 51]. In our study, most MAC isolates showed low MIC values (0.015–0.12 μg/mL) for bedaquiline, which is in agreement with previous studies [52–54]. Clinical trials are warranted to correlate the in vitro susceptibility of MAC to bedaquiline with the clinical outcome.

Cycloserine is mainly used to treat drug-resistant *M. tuberculosis*, and there are few reports on its effect on NTM. MAC isolates were completely sensitive to cycloserine in several studies [55], with an MIC breakpoint of 80 μg/mL. However, in our study, the resistant rates (≥ 64 μg/mL) are 28.9% and 42.9% for *M. intracellulare* and *M. avium*, respectively. Considering the side effects of long-term use of cycloserine and the intermediate resistance rate in vitro, it is necessary to be cautious and more data are needed to test its effect upon clinical application as a candidate drug.

In our study, the number of *M. intracellulare* isolates was much higher than that of *M. avium*, which is consistent with previous studies in China [56]. Drug susceptibilities of *M. avium* and *M. intracellulare* to several agents were different. *M. avium* had a higher resistance rate than *M. intracellulare* for clarithromycin, ethambutol, trimethoprim/sulfamethoxazole, amikacin, linezolid, clofazimine, and cycloserine. However, since the number of isolates was small in our study, most of the differences were not statistically significant, except for amikacin, ethambutol, and cycloserine. In another study in China [57], *M. intracellulare* (242 isolates) showed higher resistance rate to most drugs than *M. avium* (45 isolates), which is contrary to our results. However, no significant difference between the species was found in their study. Therefore, it is difficult to obtain significant results and provide reliable evidence for the difference in drug susceptibility of the two MAC species with a small sample size. In another study that included more strains (1883 isolates) [41], they found consistent conclusions with ours that *M. intracellulare* (1060 isolates) had lower resistant rates than *M. avium* (823 isolates) for ethambutol and amikacin. Since the two drugs are both guideline-recommended drugs for MAC, the finding is of great significance for the guidance of treatment for the two MAC species in the future. In a study in Germany [34], higher resistance rates of *M. avium* to trimethoprim/sulfamethoxazole and linezolid were also reported. In a study in Beijing in 2015 [58], the resistant rates of moxifloxacin and linezolid of the *M. intracellulare* isolates were significantly lower than that of the *M. avium*, and the resistant rate of rifampicin was lower in the *M. avium* isolates. Therefore, due to regional differences and different methods for identifying species, the results of drug susceptibility tests for *M. intracellulare* and *M. avium* varies widely across studies. Future studies are need to enrolled more MAC isolates to identify the resistance profiles in different regions.

## Conclusions

In conclusion, clarithromycin, rifabutin, amikacin, and streptomycin showed good in vitro antimicrobial activities against the MAC isolates, with resistance rates of less than 25%. However, isoniazid, rifampin, linezolid, doxycycline, and ethionamide had poor inhibitory activities, which is consistent with previous studies, and thus, not suitable to treat MAC diseases. In addition, new drugs, such as clofazimine, tedizolid, bedaquiline, and cycloserine also showed good antimicrobial activities in vitro and could be introduced to treat MAC in the future. Besides, different resistance profiles for amikacin, ethambutol, and cycloserine were seen for *M. avium* and *M. intracellulare*, but further studies are still needed to confirm these differences.

## Methods

### Study design, isolate collection and species identification

Between January 2017 and December 2020, a total of 111 MAC clinical isolates were collected from Huashan Hospital affiliated to Fudan University, Shanghai, China. They were cultured from various types of samples, including airway, blood, body fluids and soft tissues. The MAC isolates were cultured in the Middlebrook 7H9 media supplemented with 10% oleic acid/dextrose/catalase (OADC). The MAC species were identified by partial sequences of the *hsp65* and *rpoB* genes [59] and a phylogenetic tree was analyzed based on these genes. The *hsp65* gene was amplified with primers TB11 (5′-AGTTTGATCCTGGCTCAG-3′) and TB12 (5′-GGTTACCTTGTTACGACTT-3′) [60] and the *rpoB* gene was amplified with primers MycoF (5′-CGATGCGGTAAAGGTGACATTG-3′) and MycoR (5′-CCTTGACAGTGGACACCTTGGA-3′) [30]. The phylogenetic tree was built using the MEGA software version 10.0 by the Neighbor joining method with a bootstrap value 1,000. The sequences of *hsp65* and *rpoB* of MAC type strains, *M. avium subsp. avium* ATCC25291, *M. intracellulare* ATCC13950, *M. intracellulare* FLAC0133, *M. intracellulare* FLAC0181, *M. intracellulare* MOTT-02, *M. marseillense* DSM45437, *M. yongonense* 05–1390, *M. colombiense* CECT3035, *M. vulneris* DSM45247, *M. mantenii* DSM45255, *M. arosiense* DSM45069, *M. paraintracellulare* MOTT-64, and *M. intracellulare subsp. chimaera* DSM44623 were used as references.

### Drug susceptibility testing

The Sensititre Myco susceptibility plate for slow-growing mycobacteria (Thermo Fisher Scientific Inc., Waltham, MA, USA) was used to test the susceptibility of the following antimicrobial agents: clarithromycin, rifabutin, ethambutol, isoniazid, moxifloxacin, rifampin, trimethoprim/sulfamethoxazole, amikacin, linezolid, ciprofloxacin, streptomycin, doxycycline and ethionamide, according to the manufacturer protocol. The plate was designed with the reference to the CLSI document and was used in previous studies [33, 61]. Bedaquiline was purchased from AmBeed Inc. (Arlington Heights, IL, USA). Clofazimine, tedizolid and cycloserine were purchased from Aladdin (Shanghai, China). The drug susceptibility testing of bedaquiline, clofazimine, tedizolid, and cycloserine was performed using broth microdilution method according to Clinical and Laboratory Standards Institute (CLSI) protocol M24-A3. The 111 clinical MAC isolates were cultured on Middlebrook 7H11 agar for 7–14 days. *M. intracellulare* ATCC13950, *Staphylococcus aureus* ATCC29215, and *Mycobacterium smegmatis* ATCC19420 were used as controls. Then isolates were transferred to the Middlebrook 7H9 media supplemented with 10% OADC and cultured for one week at 37 °C. The bacterial suspension was adjusted to a 1 McFarland standard with sterile demineralized water and was transferred to the Middlebrook 7H9 media with 10% OADC at a ratio of 1:100. For tests using the Sensititre Myco susceptibility plate, 100 μL of the inoculum solution was added to each well of the 96-well microtitre plate containing lyophilized antibiotics. For the other four antimicrobial agents, 100 μL of both inoculum solution and serial dilutions of the agents were added to the 96-well plates. Plates were covered with adhesive seals and incubated at 37 °C in ambient air for 14 days. Results were read manually by visual growth readings according to the CLSI M24 guidelines and illustrations of various growth patterns. The minimum inhibitory concentration (MIC) values were the lowest concentrations that completely inhibited growth except for trimethoprim/sulfamethoxazole, for which the MIC value was read as the lowest concentration that inhibited 80% of the growth compared to the positive control. MIC breakpoints of the antibiotics for MAC are shown in Table 4.

**Table 4:** Breakpoints of 17 antibiotics

Antimicrobial agent	MIC breakpoints (μg/mL)		
	**Susceptibility**	**Intermediate**	**Resistance**
CLA^a^	≤ 8	16	≥ 32
RFB^b^	≤ 2	-	≥ 4
EMB^c^	-	-	> 5
INH^c^	-	-	> 0.2
MXF^a^	≤ 1	2	≥ 4
RIF^b^	≤ 1	-	≥ 2
SXT^b^	≤ 2/38	-	≥ 4/76
AMI^a^	≤ 16	32	≥ 64
LZD^a^	≤ 8	16	≥ 32
CIP^b^	≤ 1	2	≥ 4
STR^d^	≤ 16	32	≥ 64
DOX^b^	≤ 1	2–4	≥ 8
ETH^c^	-	-	> 5
TZD^e^	-	-	> 8
CFZ^d^	≤ 1	2	≥ 4
BDQ^f^	-	-	> 0.25
CS^d^	≤ 16	32	≥ 64

Notes: a, b, c denotes the breakpoints for MAC, *M.kansasii*, and *M.tuberculosis* coming from Susceptibility Testing of Mycobacteria, Nocardia, and Other Aerobic Actinomycetes; Approved Standard–Third Edition. CLSI document M24-A3. d, e, f denotes the breakpoints coming from previous studies [29, 45, 62]

*Abbreviations*: *CLA* Clarithromycin, *RFB* Rifabutin, *EMB* Ethambutol, *INH* Isoniazid, *MXF* Moxifloxacin, *RIF* Rifampin, *SXT* Trimethoprim/sulfamethoxazole, *AMI* Amikacin, *LZD* Linezolid, *CIP* Ciprofloxacin, *STR* Streptomycin, *DOX* Doxycycline, *ETH* Ethionamide, *TZD* Tedizolid, *CFZ* Clofazimine, *BDQ* Bedaquiline, *CS* Cycloserine

## Supplementary Information

**Additional file 1: Supplementary Table 1.** MIC values of the clinical MAC isolates.

## Data Availability

All data generated or analyzed during this study are included in this article and its supplementary information files.

## Abbreviations

CLSI: Clinical and Laboratory Standards Institute
IFN-γ: Interferon gamma
MAC: *Mycobacterium avium* Complex
MAC-LD: *Mycobacterium avium* Complex lung diseases
NTM: Nontuberculous mycobacteria
OADC: Oleic acid/dextrose/catalase
CLA: Clarithromycin
RFB: Rifabutin
EMB: Ethambutol
INH: Isoniazid
MXF: Moxifloxacin
RIF: Rifampin
SXT: Trimethoprim/sulfamethoxazole
AMI: Amikacin
LZD: Linezolid
CIP: Ciprofloxacin
STR: Streptomycin
DOX: Doxycycline
ETH: Ethionamide
TZD: Tedizolid
CFZ: Clofazimine
BDQ: Bedaquiline
CS: Cycloserine
